# Clinical Characteristics and Predictors of Poor Hospital Discharge Outcome for Young Children with Abusive Head Trauma

**DOI:** 10.3390/jcm8030390

**Published:** 2019-03-20

**Authors:** Chih-Chi Chen, Po-Chuan Hsieh, Carl P. C. Chen, Yu-Wei Hsieh, Chia-Ying Chung, Kuang-Lin Lin, Prevention, Protection Against Child Abuse, Neglect (PCHAN) Study Group

**Affiliations:** 1 Department of Physical Medicine and Rehabilitation, Chang Gung Memorial Hospital, College of Medicine, Chang Gung University, 5 Fuhsing St., Taoyuan 333, Taiwan; claudia5477@gmail.com (C.-C.C.); carlchendr@gmail.com (C.P.C.C.); 2 Department of Neurosurgery, Chang Gung Memorial Hospital, College of Medicine, Chang Gung University, 5 Fuhsing St., Taoyuan 333, Taiwan; pochuan.hsieh@gmail.com; 3 Department of Occupational Therapy and Graduate Institute of Behavioral Sciences, College of Medicine, Chang Gung University, 259, Sec1, WenHua First Road, Taoyuan 333, Taiwan; Ywhsieh@mail.cgu.edu.tw; 4 Healthy Aging Research Center, Chang Gung University, 259, Sec1, WenHua First Road, Taoyuan 333, Taiwan; 5 Division of Pediatric Neurology, Department of Pediatrics, Chang Gung Memorial Hospital, College of Medicine, Chang Gung University, 5 Fuhsing St., Taoyuan 333, Taiwan; 6 Study Group for Prevention and Protection Against Child Abuse and Neglect, Chang Gung Memorial Hospital, 5 Fuhsing St., Taoyuan 333, Taiwan

**Keywords:** abusive head trauma, accidental head trauma, predictors, poor outcome, Glasgow Coma Scale

## Abstract

Children with abusive head trauma tend to have worse outcomes than children with accidental head trauma. However, current predictors of poor outcomes for children with abusive head trauma are still limited. We aim to use clinical data to identify early predictors of poor outcome at discharge in children with abusive head trauma. In the 10-year observational retrospective cohort study, children aged between zero and four years with abusive or accidental head trauma were recruited. Multivariate logistic regression models were applied to evaluate factors associated with poor prognosis in children with abusive head trauma. The primary outcome was mortality or a Glasgow Coma Scale (GCS) motor component score of less than 6 at discharge. A total of 292 head trauma children were included. Among them, 59 children had abusive head trauma. In comparison to children with accidental head trauma, children with abusive head trauma were younger, had more severe head injuries, and experienced a higher frequency of post-traumatic seizures. Their radiologic findings showed common presence of subdural hemorrhage, cerebral edema, and less epidural hemorrhage. They were more in need of neurosurgical intervention. In the multivariate analysis for predictors of poor outcome in children with abusive head trauma, initial GCS ≤ 5 (versus GCS > 5 with the adjusted odds ratio (OR) = 25.7, 95% confidence interval (CI) = 1.5–432.8, *p* = 0.024) and older age (per year with the adjusted OR = 3.3, 95% CI = 1.2–9.5, *p* = 0.024) were independently associated with poor outcome. These findings demonstrate the characteristic clinical differences between children with abusive and accidental head trauma. Initial GCS ≤ 5 and older age are predictive of poor outcome at discharge in children with abusive head trauma.

## 1. Introduction

Abusive head trauma is an injury to the skull or intracranial contents of an infant or child younger than five years caused by inflicted blunt impact and/or violent shaking [1]. Abusive head trauma is a major cause of physical child abuse fatalities [2]. Studies of consecutively admitted infants and preschoolers with head injuries report rates of abusive head trauma ranging from 4% to 24% [3], with greater incidence in children younger than one year [4]. The biomechanical forces generated at the time of brain injury differ in abusive versus accidental head trauma [3]. Abusive head trauma is often associated with significant rotational acceleration-deceleration force, which seldom occurs in accidental head injury. With different etiology and mechanism, children with abusive head trauma tend to have different presentations and characteristics from those with accidental head trauma. Children with abusive head trauma are slightly more often boys, often younger than one year, commonly with impaired consciousness levels when visiting emergency departments, of higher frequency in seizure occurrence, and are often patients with increased rates of retinal hemorrhage and more subdural hematoma (SDH) in neuroimaging findings [5,6]. Other associated injuries include certain extra-cranial fractures [7]. Moreover, cutaneous and visceral injuries are more common in abusive head injury cases than in those due to accidental head injury [6]. Understanding the characteristics of abusive head trauma can help alert clinicians to the correct diagnosis and the need for a thorough diagnostic evaluation [6,8,9].

Predictors of poor outcome for children with head trauma have been studied [8,10,11,12,13,14,15,16]. However, studies of predictors of poor outcomes in abusive head trauma cases are still limited even though it is known that children with abusive head trauma tend to have worse outcomes than children with accidental head trauma [3,8,17,18,19]. A prior multicenter retrospective study identified risk factors for mortality of children with abusive head trauma and included low Glasgow Coma Scale (GCS), retinal hemorrhage, parenchymal hemorrhage, and cerebral edema [5]. Besides higher mortality, about 42%–96% survivors suffered neurologic morbidity when discharged, and needed more long-term care [5].

A recent long-term follow-up study suggested that young children with pediatric traumatic brain injury that were discharged with an unfavorable status were associated with a higher probability of a continuing unfavorable outcome [20]. Predicting short-term poor outcome for young children with abusive head trauma helps clinicians, patients, and their families not only in knowing the prognosis at discharge but also in determining the appropriate long-term care preparations. In this study, we compared clinical differences in presentations of children with abusive versus accidental head trauma and determined predictors of short-term poor outcome (mortality or motor component of GCS < 6 at discharge) in children with abusive head trauma. Our overall goal was to provide insight into the different presentations of abusive versus accidental head trauma and improve prognostic counseling and care for children with abusive head trauma.

## 2. Materials and Methods

### 2.1. Study Design and Selection of Patient Cohort

This is a retrospective, observational cohort study conducted in a 3000-bed medical center in north Taiwan for children aged between zero and four years admitted from January 2005 to December 2014 with traumatic head injury. Patients were eligible if they were assigned International Classification of Diseases, Ninth Edition (ICD-9) (WHO 1977) diagnostic codes 850–854 for intracranial injury. Each medical record was reviewed by a physician and a research nurse who categorized patients into abusive head injury or accidental head injury according to the discharge diagnoses of the patients. Any disagreement would be discussed and the medical charts would be reviewed to confirm the final diagnosis. Accidental head trauma patients were defined as patients that suffered from traffic accidents, witness accidents, and isolated incidents (falls from porches, upper-story windows, or falls as an infant walker) [6]. Diagnosis of abusive head trauma was made by the Child Abuse Team in our hospital and by the attending physicians of the patients if any admissions were given by the caregiver of the child or any eyewitness. In addition, if the primary caregiver of the child provided an explanation that was inconsistent with the developmental capabilities of the child or inconsistent with severity of the physical findings, as well as in the case of children with more than two non-cranial injuries considered moderately or highly specific to abuse including (1) bruising of the torso, ear, or neck, (2) fractures of the ribs, metaphyseal regions, or long bones, and (3) intraocular injury with signs of retinal hemorrhage, vitreous hemorrhage, disc swelling, macular retinoschisis, macular hole, or retinal detachment, they would be diagnosed with abusive head trauma [18,21,22]. In this study, we only analyzed abusive head trauma from physical abuse. Patients that died at the scene or during transportation were excluded. Patients with prior central nervous system disorders or any other disorders involving motor deficit, seizure, cognition deficit, emotional problems, or developmental delay before their injury were also excluded due to difficulty in assessing the study outcome. The according histories were gathered and reviewed during admission and noted in patient charts. Those also excluded were patients whose primary injury was extra-cranial despite also carrying an ICD-9 code for intracranial injury. This study was approved by the Institutional Review Board in Chang Gung Memorial Hospital.

### 2.2. Variable Definitions

Informative data were extracted from the medical records of all eligible subjects. Parameters selected for evaluation of potential associations with poor neurologic prognosis included gender, age, injury severity, intracranial computed tomography (CT) findings in the emergency department, instances of post-traumatic seizure, and whether neurosurgery was conducted or not.

Injury severity was stratified by initial GCS after resuscitation. We used the pediatric GCS score for preverbal children and standard GCS score for children who had verbal interaction before head trauma [23,24,25]. The GCS, which ranges from 3 to 15, was scored by emergency medical service personnel at the emergency department, at admission regular intervals. The initial GCS score, which was required to persist for 6 h, was defined as the first post-resuscitation GCS score obtained at the emergency department [26]. The 6-h criterion was used to establish central neurologic impairment of a non-transient nature [27]. Post-traumatic seizures were defined as seizures occurring in the first week after traumatic brain injury, and were considered to be provoked by trauma [28].

Initial non-contrast CT scans obtained at the emergency department were reviewed by pediatric radiologists during admission. Specific types of intracranial injuries including cranial bone fracture, subdural hematoma (SDH), epidural hematomas (EDH), parenchymal hemorrhage, subarachnoid hemorrhage (SAH), brain infarction, cerebral edema, and mass effect were identified [29,30].

The motor component of the GCS ranges from 1 to 6. Poor outcome was defined as in-hospital mortality or inability of patients to obey simple commands (motor component of GCS less than 6) at discharge. Good outcome was defined as a GCS motor component score of 6 for children when they were discharged from the hospital.

### 2.3. Patients Selections

Of the 316 children aged between zero and four years initially identified for further study, four were excluded for having a final diagnosis other than head trauma, six were excluded due to a previous central nervous system disease (one central nervous system leukemia, four brain operation history, and one brain tumor), two were excluded for having a primary extra-cranial injury, two died before admission to emergency room, and 10 were excluded for incomplete data recording. The resulting study sample consisted of 292 cases, including 59 (16.3%) with abusive head trauma and 233 with accidental head trauma.

### 2.4. Statistics

Descriptive statistics were used to identify differences in clinical characteristics and outcome variables among patients with abusive versus accidental head trauma. For categorical variables, comparisons were made using Pearson’s chi-square tests or Fisher exact test if any expected cell size was less than 5. The Mann–Whitney U test was used to compare the continuous age distribution of patients with abusive and accidental head trauma. To identify potential predictors of poor outcome among children with abusive head trauma, multivariate logistic regression analyses were conducted. The candidate predictors with a significant level of *p* < 0.1 were identified by univariate analysis. A *p*-value < 0.05 was considered statistically significant or the multivariate analyses. We used the receiver operating characteristic (ROC) to evaluate the ability to predict poor outcome by the identified potential predictors in multivariate analysis. All statistics were performed using STATA version 14.0 software (STATA, Inc., College Station, TX, USA).

## 3. Results

### Demographics

Table 1 provides the demographic and clinical characteristics of participants with abusive and accidental head trauma. Compared to children with accidental head trauma, children with abusive head trauma were younger (*p* < 0.001), had more severe injury severity, which presented as a lower initial GCS score (*p* < 0.001), and had higher frequency of post-traumatic seizure (*p* < 0.001). Their intracranial radiology findings have more common SDH (*p* < 0.001), cerebral edema (*p* = 0.004), and less EDH (*p* = 0.002). Children with abusive head trauma were more in need of neurosurgical intervention (*p* < 0.001). Their outcomes were poorer, with about 15% children suffering mortality or a GCS motor component less than 6 when discharged from hospital. No significant differences in gender distribution, CT evidence of skull fracture, parenchymal hemorrhage, SAH, brain infarction, or mass effect were detected between two groups of children.

Table 2 shows the results of univariate analyses concerning potential clinical predictors associated with poor outcome in children with abusive head trauma. Older age (*p* = 0.004), decreased GCS score (*p* < 0.001), and presence of SAH were associated with a poor outcome (*p* = 0.027). There were no associations between poor outcome and gender, seizure, cranial bone fracture, SDH, EDH, parenchymal hemorrhage, infarction, cerebral edema, mass effect, or neurosurgical intervention.

In multivariate analyses (Table 3), gender, age, GCS ≤ 5, EDH, and SAH were included. Children with initial GCS ≤ 5 had increased incidence of poor outcome compared to children with better initial GCS (odds ratio (OR) 25.7, 95% confidence interval (CI) = 1.5–432.8). Age was another independent predictor of poor outcome. With each year older in age, the odds of poor outcome were 3.3 times higher (95% CI = 1.2–9.5). The area under the curve for the multivariate model including age and GCS ≤ 5 variables was 0.838.

## 4. Discussion

Our results showed that, in comparison with accidental head trauma, children with abusive head trauma had younger age, more severe head injury severity, and higher frequency of post-traumatic seizures. The radiologic findings in abusive head trauma also had a higher presence of SDH and cerebral edema, and less-common EDH. Children with abusive head trauma were more in need of neurosurgical intervention and had poorer outcome than children with accidental head trauma. Regarding prognosis, an initial GCS of 3–5 is a strong independent risk factor of poor outcome in children with abusive head trauma. Older age is another identified predictor of poor outcome in children with abusive head trauma aged between zero and four years, while older age predicted a poorer outcome. 

Demographic characteristic including age and gender were evaluated in this study. In both abusive and accidental head trauma, about 60% of the children were boys. Such a finding is compatible with a recent study which also showed that boys are slightly more commonly victims of abusive head trauma [31]. Gender did not influence the outcome in children with abusive head trauma. Such findings are compatible with previous studies which all showed no difference in outcome for both abusive and accidental head trauma [8,18,32]. In our study, 90% of children with abusive head trauma were under one year of age while only 60% of children under one year of age had sustained accidental head trauma. Children with abusive head trauma with poor outcomes were significantly older than those with good outcomes. Such findings are compatible with a recent 20-year large cohort study, which also found that the mortality of patients with abusive head trauma was higher in children over two years of age than under two years of age [9]. Our study further found that increased age was an independent predictor for poor outcome in multivariate analysis. Such results are different when children sustain accidental head trauma, with a recent study finding that children being less than two years old was an independent predictor of mortality in children with severe head trauma [15]. Older children that suffer abusive head trauma may have different mechanisms of trauma than younger children. They might be punched, kicked, or thrown, which has a higher risk of a poor outcome [9]. Older children may also have suffered repetitive unrecognized head trauma and delayed transfer until it became life threatening. Previous literature presented that older age is a risk for missed cases of abusive head trauma, which supports this finding [33].

Our previous study of a series of 309 children with traumatic brain injury suggested that GCS 5 is a critical predictive score for poor outcome [27]. In current this study, 13.5% of children with abusive head trauma had initial GCS ≤ 5, while only 3.4% of children in accidental head trauma had initial GCS ≤ 5. Children with abusive head trauma had significantly higher injury severity than children with accidental head trauma. This study further proved that initial GCS ≤ 5 was a strong independent predictor of poor outcome. Although it is still debated whether or not GCS alone is detailed enough for detecting tiny neurologic change [34], our study supports GCS as an accurate predictor of injury outcome in abusive head trauma patients, as presented by another study [35].

Post-traumatic seizure can increase risk of childhood-onset epilepsy, and even many years later [36]. According to previous studies, a higher percentage of children with abusive head trauma presented with post-traumatic seizure [4]. Our study did not yield evidence that post-traumatic seizure was associated with short-termed poor outcome in children with abusive head trauma.

With regard to neuro-radiologic findings, our study confirmed results similar to those of previous studies indicating that SDH and brain edema are more common in abusive head trauma, whereas EDH is more common in accidental head trauma [37]. This may be explained by differences in inertial force resulting from abusive behavior such as violent shaking, leading to the tearing of bridging cortical veins and subsequent SDH. On the other hand, the direct impact to the skull associated with accidental head trauma may result in higher rates of EDH [38]. There was no association of SAH with abusive head trauma in our study, but for children with abusive head trauma, those with SAH were associated with poor outcome when discharged from the hospital. In shaking injury, SAH commonly results due to tears of the small vessel in the pia and arachnoid mater and appears in the interhemispheric fissure or high convexity [39]. Hochstadter et al. retrospectively analyzed initial CT head imaging within 24 h of hospital admission in 171 severe traumatic brain injury patients and obtained similar findings which suggested that the presence of SAH was indicative of higher pediatric traumatic brain injury severity and a higher level of required care after hospital discharge. SAH was not independently associated with increased risk of mortality [40]. SAH was also not an independent factor leading to poor outcome in our study.

A higher percentage of children with abusive head trauma needed neurosurgical intervention, which may reflect the greater severity of abusive head trauma and medical demand compared to accidental head trauma. Most children with abusive head trauma receive operations such as subdural hemorrhage drainage or craniotomy [38]. For these children with an indication of neurosurgical intervention, their prognosis is the same with those without intervention in our study.

### Limitations

This study has some limitations. First, our results were based on a hospital-based registry, limiting the generalizability of the sample population. Further prospective studies with larger sample sizes would be more representative of the population. Second, not all potential risk factors for poor outcome were examined in this study, as we only assessed routinely documented and well-recorded clinical characteristics in our study. The purpose of this study design was to find out predictors of poor outcome from these commonly known and easily available initial measurements from emergency departments. Third, some unidentified abusive head trauma may have been incorrectly categorized as accidental head trauma. Our results showed the differences in initial characteristics of children with abusive versus accidental head trauma and provided more information for clinical clinicians to identify abusive head trauma. Furthermore, although we noted that age is an independent factor for outcome determination, the small number of the patients older than one year of age showed a risk of increase in the possibility of bias. Moreover, the type of abuse might affect the final prognosis; however, our institute did not record this data in detail in the early years. We cannot obtain these data to make an adequate analysis, which is another limitation of our study. The prospective collection might help us understand the significance of abuse type in the future. Some manuscripts discuss outcomes of pediatric traumatic brain injury using the Glasgow Outcome Scale-Extended Pediatric Revision (GOS-E Peds) [41]. Our project began in 2005, so we lack the data of GOS-E Peds from earlier patients. We recorded patients’ outcomes including death and motor component of GCS at discharge and used death and a motor component of GCS less than 6 as our poor outcome. Finally, long-term outcome was not assessed in this study due to the limit of the retrospective study. Further long-term, prospective cohort studies involving more identified risk factors are warranted to understand the clinical presentation, outcomes, and prognostic factors for abusive head trauma.

## 5. Conclusions

In this study of children with head trauma aged between zero and four years, abusive head trauma was associated with worse outcome and had different characteristics compared to accidental head trauma. Children with abusive head trauma were younger, and presented with lower initial GCS, higher frequency of post-traumatic seizure, more common intracranial radiologic findings, more need for neurosurgical intervention, and poorer outcomes at discharge. Initial GCS ≤ 5 and older age were found to be independent predictors of poor outcome at discharge. For older children with suspected abusive head trauma and GCS ≤ 5, clinicians should be aware of higher mortality risk, higher frequency of unconsciousness at discharge, and long-term care needs.

## Figures and Tables

**Table 1:** Demographic and clinical characteristics of all children with abusive and accidental head trauma.

	Abusive Head Trauma (HT) (*n* = 59) *n* (%)	Accidental HT (*n* = 233) *n* (%)	*p* Value
**Clinical Characteristics**			
**Gender**			
Boys	37 (62.7)	137 (58.8)	0.584
Girls	22 (37.3)	96 (41.2)	
**Age (years)**			
Median (25, 75%)	1 (1, 2)	3 (1, 5)	<0.001
**Glasgow Coma Scale (GCS) Score**			
13–15	29 (49.2)	198 (85.0)	<0.001
9–12	12 (20.3)	12 (5.1)	
6–8	10 (16.9)	15(6.4)	
3–5	8 (13.5)	8 (3.4)	
**Seizure disorder**			
Present	43 (72.9)	48 (20.6)	<0.001
Not present	16 (27.1)	185 (79.4)	
**Cranial bone fracture**			
Present	3 (5.1)	12 (5.2)	0.642
Not present	56 (94.9)	221 (94.8)	
**Subdural hemorrhage**			
Present	46 (78.0)	80 (34.3)	<0.001
Not present	13 (22.0)	153 (65.7)	
**Epidural hemorrhage**			
Present	3 (5.1)	48 (20.6)	0.002
Not present	56 (94.9)	185 (79.4)	
**Parenchymal hemorrhage**			
Present	3 (5.1)	11 (4.7)	0.564
Not present	56 (94.9)	222 (95.3)	
**Subarachnoid hemorrhage**			
Present	14 (23.7)	25 (10.7)	0.009
Not present	45 (76.3)	208 (89.3)	
**Brain infarction**			
Present	2 (3.4)	3 (1.3)	0.266
Not present	57 (96.6)	230 (98.7)	
**Cerebral edema**			
Present	6 (10.2)	5 (2.2)	0.004
Not present	53 (89.8)	228 (97.8)	
**Mass Effect**			
Present	6 (10.2)	11 (4.7)	0.110
Not present	53 (89.8)	222 (95.3)	
**Neurosurgical intervention**			
Yes	35 (59.3)	53 (22.8)	<0.001
No	24 (40.7)	180 (77.2)	
**Mortality**			
Yes	3 (5.1)	6 (2.6)	0.9926
No	56 (94.9)	227 (97.4)	
**Poor outcome**			
Yes	9 (15.3)	8 (3.4)	0.001
No	50 (84.7)	225 (96.6)	

**Table 2:** Univariate analyses of poor-outcome-predictor association in children with abusive head trauma.

	Poor Outcome *n* (%)	Good Outcome *n* (%)	Test Statistic	*p* Value
**Gender**				
Male	3 (8.1)	34 (91.9)	3.92 *	0.066
Female	6 (27.3)	16 (72.7)		
**Age (years)**				
Median (25, 75%)	2 (1, 2.5)	1 (1, 1)	−2.89 ^#^	0.004
**GCS**				
12–15	0 (0)	31 (100)	19.37 *	<0.001
9–11	0 (0)	10 (100)		
6–8	4 (40.0)	6 (60.0)		
3–5	5 (62.5)	3 (37.5)		
**Severe head injury**				
GCS ≤ 5	5 (62.5)	3 (37.5)	15.98 *	0.001
GCS >5	4 (7.8)	47 (92.2)		
**Seizure**				
Present	7 (16.3)	36 (83.7)	0.13 *	0.537
Not present	2 (12.5)	14 (87.5)		
**Cranial Bone Fracture**				
Present	1 (33.3)	2 (66.7)	0.80 *	0.397
Not present	8 (14.3)	48 (85.7)		
**Subdural hemorrhage**				
Present	7 (15.2)	39 (84.8)	<0.01 *	0.642
Not present	2 (15.4)	11 (84.6)		
**Epidural hemorrhage**				
Present	2 (66.7)	1 (33.3)	6.46 *	0.058
Not present	7 (12.5)	49 (87.5)		
**Parenchymal hemorrhage**				
Present	0 (0.0)	3 (100.0)	0.21 *	1.000
Not present	9 (16.1)	47 (83.9)		
**Subarachinoid hemorrhage**				
Present	5 (35.7)	9 (64.3)	5.94 *	0.027
Not present	4 (8.9)	41 (91.1)		
**Infarction**				
Present	0 (0)	2 (100.0)	0.63 *	0.716
Not present	9 (15.8)	48 (84.2)		
**Cerebral edema**				
Present	2 (33.3)	4 (66.7)	1.69 *	0.224
Not present	7 (13.2)	46 (86.8)		
**Mass Effect**				
Present	1 (16.7)	5 (83.3)	0.01 *	0.647
Not present	8 (15.1)	45 (84.9)		
**Neurosurgical intervention (%)**				
Yes	5 (14.3)	30 (85.7)	0.06 *	0.540
No	4 (16.7)	20 (83.3)		

*: Chi Square Statistic; #: Z test.

**Table 3:** Multivariate analysis examining the predictor of poor outcome after discharge of children with abusive head trauma.

	Adjusted Odds Ratio (OR)	95% Confidence Interval (CI)	*p* Value
Gender	0.098	0.01–1.28	0.077
Age	3.342	1.17–9.52	0.024
GCS ≤ 5	25.71	1.53–432.78	0.024
Epidural hemorrhage	2.377	0.02–314.77	0.728
Subarachnoid hemorrhage	9.872	0.83–117.49	0.070

GCS = Glasgow Coma Scale; OR = odds ratio; CI = confidence interval.
